# Development of the University Food Environment Assessment (Uni-Food) Tool and Process to Benchmark the Healthiness, Equity, and Environmental Sustainability of University Food Environments

**DOI:** 10.3390/ijerph182211895

**Published:** 2021-11-12

**Authors:** Davina Mann, Janelle Kwon, Shaan Naughton, Sinead Boylan, Jasmine Chan, Karen Charlton, Jane Dancey, Carolyn Dent, Amanda Grech, Victoria Hobbs, Sophie Lamond, Sandra Murray, Melissa Yong, Gary Sacks

**Affiliations:** 1 Global Obesity Centre, Institute for Health Transformation, Deakin University, Geelong, VIC 3220, Australia; d.mann@deakin.edu.au (D.M.); Janelle.l.kwon@gmail.com (J.K.); shaan.naughton@deakin.edu.au (S.N.); jasmine.chan@deakin.edu.au (J.C.); Victoria.hobbs@deakin.edu.au (V.H.); 2 Charles Perkins Centre, School of Life and Environmental Sciences, University of Sydney, Sydney, NSW 2006, Australia; sinead.boylan@sydney.edu.au (S.B.); Amanda.grech@sydney.edu.au (A.G.); 3 School of Medicine, University of Wollongong, Wollongong, NSW 2522, Australia; karenc@uow.edu.au; 4 Buildings and Property Division, Monash University, Clayton, VIC 3800, Australia; jane.dancey@monash.edu.au; 5 Nutrition & Dietetics, College of Nursing & Health Sciences, Flinders University, Bedford Park, Adelaide, SA 5042, Australia; Carolyn.dent@flinders.edu.au; 6 Melbourne Law School, Melbourne School of Government, University of Melbourne, Carlton, VIC 3053, Australia; sophie.lamond@unimelb.edu.au; 7 School of Health Science, University of Tasmania, Launceston, TAS 7250, Australia; Sandra.murray@utas.edu.au; 8 Division of Student Life, Deakin University, Burwood, VIC 3125, Australia; melissa.yong@deakin.edu.au

**Keywords:** food environments, environmental sustainability, assessment, tool, equity, university

## Abstract

Globally, there is increasing interest in monitoring actions to create healthy, equitable and environmentally sustainable food environments. Currently, there is a lack of detailed tools for monitoring and benchmarking university food environments. This study aimed to develop the University Food Environment Assessment (Uni-Food) tool and process to benchmark the healthiness, equity, and environmental sustainability of food environments in tertiary education settings, and pilot test its implementation in three Australian universities in 2021. The Uni-Food tool development was informed by a review of the literature and input from an expert advisory panel. It comprises three components: (1) university systems and governance, (2) campus facilities and environments, and (3) food retail outlets. The process for implementing the tool is designed for universities to self-assess the extent to which they have implemented recommended practice in 68 indicators, across 16 domains, weighted based on their relative importance. The pilot implementation of the tool identified moderate diversity in food environments across universities and highlighted several opportunities for improvements at each institution. The assessment process was found to be reliable, with assessors rating the tool as easy to use, requiring minimal resources. Broad application of the tool has the potential to increase accountability and guide best practice in tertiary education and other complex institutional settings.

## 1. Introduction

Food environments, including the places where people access food, the types of foods available, and the ways foods are marketed, have an enormous impact on population diets [1,2,3]. Currently, contemporary food environments are characterised by the high availability and heavy promotion of ultra-processed foods that are often high in salt, saturated fats, added sugar and energy [2,3]. These unhealthy food environments are recognised as a major driver of unhealthy diets that are a leading contributor in preventable disease burdens globally [4]. They have also contributed to substantial inequalities in diet-related health outcomes, with people from lower socio-economic groups, minority groups, and living in remote areas more likely to have a diet of poorer quality [5,6]. 

Food systems can also present risks to planetary wellbeing, with current food systems contributing substantially to climate change, biodiversity loss, depletion of freshwater resources and pollution of waterways [1,7,8]. Dietary patterns that contain high levels of red and highly-processed meats and ultra-processed foods present risks to human health and typically have a higher environmental impact than dietary patterns higher in whole foods and plant-based foods [9,10]. To reduce diet-related risks to human and planetary health, food systems and dietary habits will require a major transformation to become more healthy, equitable and environmentally sustainable which will require comprehensive action across multiple settings to bring about the necessary change [1,7,11].

Globally, there is increasing interest in monitoring actions to create healthy, equitable and environmentally sustainable food environments [12]. Monitoring and benchmarking food environments can increase accountability for action by facilitating engagement with decision makers, identifying and communicating recommended actions, and highlighting priority areas for change [13]. Several initiatives have been developed aimed at monitoring and benchmarking a diverse range of food environments [14,15]. The International Network for Food and Obesity/noncommunicable disease Research, Monitoring and Action Support (INFORMAS), currently active in more than 55 countries, is a global network that aims to monitor and benchmark food environments using standardised global approaches, tailored to the local context [13,16]. The activities of INFORMAS have been successful in building capacity and supporting increased policy action for improving the healthiness of food environments [13,17]. However, thus far INFORMAS has not included a focus on environmental sustainability [13]. Moreover, most of the tools used by INFORMAS do not integrate multiple components of food environments as part of the assessment process. In settings with complex food environments, with multiple food outlets and a range of influences on food consumption (such as the university campus environment), a comprehensive assessment, including composite measures of healthiness and environmental sustainability, is needed to inform and prioritise required actions [18].

University campuses are complex food environments catering to diverse communities. Campus food environments typically include a large number and wide range of food retail outlets, vending machines, student residences, food provision and catering services, food co-operatives, institutional and student-run events and promotional activities that cater to, and influence, the food choices of large numbers of students and staff [19,20]. Many university campuses also include gardens, food production for curriculum and research activities, and welfare services. In Australia, the majority of student populations live off-campus, and typically do not include institutionally-run dining halls or the provision of meal plans, instead relying on largely private businesses to sell food to the university community [20]. University food environments are an important setting to target as part of efforts to improve population diets in Australia as they cater to over 1.4 million students, with a further 259,100 full-time equivalent employees [21]. 

Universities are a promising setting for developing, testing and promoting strategies and action to transform food environments [22]. Historically, the tertiary education sector has been a leader in innovation and social change through its capacity to develop and enact policy, mobilise communities, develop infrastructure and direct relatively large levels of expenditure towards particular goods and services [23,24]. Further, they can educate students as future citizens, and use their campuses to model and test novel ideas [24,25,26].

Whilst numerous initiatives have been established within the tertiary education sector in an attempt to create healthy and environmentally sustainable campus environments, there is a noticeable gap in the development of tools for monitoring and benchmarking food environments of university campuses. At a global level, the Okanagan Charter for Health Promoting Universities and Colleges (the Okanagan Charter) developed in 2015 by tertiary education and health promotion delegates from over 45 countries, provides an action framework for tertiary education institutions to embed health into all aspects of campus culture and to lead health promotion action locally and globally. While the Okanagan Charter recognises the importance of healthy, supportive environments, it makes no specific mention of food environments [27]. The Sustainability Tracking, Assessment and Rating System (STARS) is an accreditation scheme that was established in the U.S.A. in 2009 to help tertiary education institutions measure and improve their sustainability performance, and has over 1000 registered institutions globally [28]. STARS measures tertiary education institutions in six main categories, including academic programming, community engagement, operations, governance and innovation, with assessment broken down into 69 credit points featuring several hundred individual reporting fields. Within these, only two credit points are specifically dedicated to food environments, with food covered briefly in areas such as purchasing, waste management and innovation [28]. 

To date, there have only been a small number of assessments of the healthiness of campus food environments [29,30,31,32,33]. None of these studies have comprehensively assessed the healthiness, equity and environmental sustainability of university food environments, and no detailed frameworks exist to guide policy and practice in the area. To address this gap, this study aimed to: (1) develop the University Food Environment Assessment (Uni-Food) tool to benchmark the healthiness, equity, and environmental sustainability of food environments in tertiary education settings; (2) develop a process for implementation of the Uni-Food tool; and (3) pilot test the ease of implementation and reliability of the Uni-Food tool and process. The goal of the study was to contribute to the work of INFORMAS by: increasing accountability of the tertiary education sector for creating healthy, equitable and environmentally sustainable campus food environments; supporting universities in identifying areas for action; paving the way for the further development of tools for monitoring and benchmarking other settings with complex food environments. 

## 2. Methods

To address the aims of this paper, the study consisted of three distinct methodological processes which are described within this section: (1) the methods of development of the Uni-Food tool; (2) the methods of development of the process for implementation of the Uni-Food tool; and (3) the methods used for the pilot implementation of the tool and process in three Australian universities in 2021. 

### 2.1. Development of the Uni-Food Tool 

Development of the Uni-Food tool took place over the period of 18 months (October 2019–March 2021). An iterative process was employed to develop the tool whereby existing literature was used to inform a first draft of the tool that was then gradually refined through repeated review by an Australian national expert advisory panel consisting of members (*n* = 15) with expertise in a range of areas relevant to campus food environments, including public health and health promotion, campus operations and facilities, and relevant areas of research and teaching. The research team and expert advisory panel involved in the development of the Uni-Food tool represented seven universities across Australia, and various roles within the university ranging from research professors, senior lecturers and campus facility managers and members.

For the purposes of the study, healthy, equitable and environmentally sustainable food environments were conceptualised as food environments in which: (1) a broad range of culturally-diverse healthy and environmentally sustainable foods and beverages were readily available and affordably priced; (2) foods and beverages that did not support healthy and environmentally sustainable diets (such as sugary drinks, deep-fried foods, chips and confectionery) were not readily available and were not promoted, with pricing strategies that disincentivised their consumption; (3) students and staff were supported to adopt healthy and environmentally sustainable diets, including through the facilities made available, and clear and readily understandable information provided in food retail outlets (e.g., through product and shelf-labelling). Healthy foods and beverages were defined based on the Australian Dietary Guidelines [34] and other relevant Australian state and territory government guidelines [35]. In operationalising the concept of environmentally sustainable foods and beverages, the focus was on reducing the environmental impact of food sold and consumed on campus. This includes minimising food waste and food packaging, preferencing fresh, minimally processed food and locally sourced food produced in season, and limiting red and processed meat [7]. In operationalising the concept of ‘equity’, the focus was on access to, and affordability of, culturally diverse healthy and environmentally sustainable foods and beverages.

To identify literature to inform the tool, we conducted a search of peer-reviewed papers that described and/or applied tools designed to assess the healthiness, equity and/or environmental sustainability of university campuses or food environments more generally. A selection of grey literature was also included based on the advice of the expert advisory panel. Twelve relevant tools were identified, which are summarised in Table S1 in the Supplementary File. We also considered literature that described barriers and enablers of successful actions to improve food environments in multiple settings, including universities, as well as studies that provided relevant evidence of the effectiveness and cost-effectiveness of interventions for improving the healthiness, equity and/or environmental sustainability of food environments. These studies were identified by members of the expert advisory panel. For each tool identified in the literature, all of the indicators within the tool were assessed for inclusion in the Uni-Food tool based on relevance to the study objectives, and suitability to the Australian university setting. Further indicators from the literature on broader settings were included if they were relevant to the university environment and had demonstrated evidence of effectiveness or were identified as best practice by the expert advisory panel. Shortlisted indicators were refined to combine any similar indicators, modified for the Australian university context, and conceptually aggregated into components, domains, and sub-domains. The identification and refinement for each of the tool’s components, domains, sub-domains, and indicators were achieved via the expert advisory panel reaching consensus through discussion. The tool was designed to be readily modified to relevant healthy food guidelines applicable in each state and territory across Australia [36].

For each indicator included in the tool, we defined a best practice statement and assessment criteria using a graded points scheme. While the assessment criteria varied for each indicator, a university would typically receive the maximum of 10 points for a particular indicator if the level of implementation of relevant policies or practices matched the best practice statement. Relatively high scores would be allocated if the implementation of relevant policies and practices was comprehensive, routinely monitored and reported, and applied across all relevant university settings (e.g., all campuses). Lower scores would be allocated if the level of implementation fell short of best practice, with the scoring designed to give universities credit for all aspects of implementation (including planning underway for relevant action, informal action or relevant pilot implementation). Good practice examples (internationally and nationally) in relation to each domain were identified based on a review of the literature and consultation with the expert advisory panel. 

A preliminary version of the tool was developed following an iterative review by the expert advisory panel between April–November 2020. Modifications throughout this process included amendments to the scope of individual indicators, refinements to the assessment criteria, addition of new indicators, and changes to the conceptual aggregation of indicators. Relative weighting of the components, domains and sub-domains within the Uni-Food tool were determined based on their relative importance, through consultation with the expert advisory panel involving an online survey in which each member of the expert advisory panel rated the importance and proposed weighting for each component, domains, and sub-domains of the tool. Results from this survey were aggregated and relative weightings calculated. 

### 2.2. Development of the Process for Implementing the Uni-Food Tool

The process for implementing the Uni-Food tool was modelled on the process for implementation of other benchmarking tools developed by INFORMAS. These tools were the Healthy Food Environment Policy Index (Food-EPI), applied in more than 15 countries [31,37], and the BIA-Obesity (Business Impact Assessment—obesity and population-level nutrition), applied in five countries [14]. These tools largely focused on assessment of individual components of food environments, such as food composition, food promotion, food prices and government policies to support healthy food environments [13,16]. We adapted the process for application in the university context, including incorporation of detailed data on the level of implementation of policies and related practices.

The application of the tool was designed for self-assessment by universities, supported by a team familiar with application of the tool, as part of the INFORMAS network. The process was designed to be collaborative, including engagement with various stakeholders of the university, including campus facilities and services, academic staff, and student representatives. The process was also designed to require minimal time and resources to implement, and to be easily and readily applied across multiple campuses varying in size and number of food retail outlets and completed by auditors who may not have detailed knowledge in nutrition or food environments. The tool was designed for repeated application (approximately every two years) at each university to measure progress over time.

### 2.3. Pilot Implementation of the Uni-Food Tool and Process

After completion of the development of the Uni-Food tool, in 2020, we conducted a pre-pilot at one Australian university to test and refine the Uni-Food assessment process and refine data collection and analysis templates (using Microsoft Excel) and processes [38].

During the first 6 months of 2021, the Uni-Food tool was piloted across three universities (not including the pre-pilot university) within Australia to: (1) assess the feasibility of implementation of the tool and its application process; (2) test for face-validity and inter-rater reliability of the tool and (3) assess the participating universities food environments. Universities were selected with purposeful sampling to ensure diversity in the location of the universities across Australia and campus size. Appropriate universities were identified through contacts of the expert advisory panel. The three universities selected for pilot testing were located across three different states within Australia (New South Wales, Queensland, and South Australia). The number of food retail outlets at each university ranged between 10 and 21.

Data collection for each university were conducted by two students currently enrolled in university research placement units and were supervised by academic members of staff (the university project team). Students were trained (via video conference) by members of the team that had developed the tool (the research team) in the use of the tool prior to data collection. Data collected from each university was cross-checked by members of the research team to ensure data collection was complete and substantiated by evidence (e.g., relevant universities documents and photo evidence of the relevant campus environment). Where necessary, additional information was sought from the university project team. Two independent members of the research team, both of whom were familiar with the tool, assigned scores for each indicator for each of the three universities based on the data collected. In instances where discrepancies between the two scores were evident, the assessment was discussed with a third member of the research team to assign an agreed score. Overall scores for each university were calculated according to the agreed scores for individual indicators and relevant indicator weightings. The research team and each university project team reviewed the final scores for face validity. Gwet’s AC1 (unweighted) integrated reliability coefficients (calculated using Rstudio) were used to calculate the inter-rater reliability for each university assessment score [39].

To understand assessors’ experience using the Uni-Food tool, approximately two weeks after the completion of data collection, student members of the university project teams completed a short (5–10 min) online survey via Qualtrics. The survey consisted of both open-ended and Likert-scale questions and collected information on the Uni-Food tool’s usability, the usefulness of the online training received, resources required to complete the process, and participants’ overall experience in using the tool. Meetings with the project team leader from each university were conducted to understand general experiences with implementing the tool and process, and resources required (including time, cost, and personnel). 

## 3. Results

### 3.1. Uni-Food Tool

The Uni-Food tool consists of 68 indicators across 3 components: (1) university systems and governance, (2) campus facilities and environments, and (3) food retail outlets. Within each component, indicators are further categorised into 16 domains and 42 sub-domains as shown in Table 1. The contribution of each component, domain, and sub-domain to a university’s overall score (out of 100) is based on their assigned weighting (refer to Table S2), with the ‘university systems and governance’ and ‘campus facilities and environment’ components accounting for 40% each and the ‘food retail environment’ component accounting for 20%. The indicators within each sub-domain, and related assessment criteria, are provided in Table S3. A university’s overall score out of 100 is designed to reflect the extent of implementation of best practice (0–25%: very little; 25–50%: low; 50–75%: medium; and 75–100%: high).

The ‘university systems and governance’ component includes indicators that assess: university leadership and planning; strategies and policies; monitoring and reporting on key food environment characteristics and outcomes; funding and resources allocated to support food environment activities; engagement of staff and students in the design of food environments. The ‘campus facilities and environment’ component includes indicators that assess physical and social environments on campus (excluding in food retail outlets that are assessed in a separate component). Domains within this component relate to programs and facilities that promote the availability, accessibility, and affordability of healthy, equitable and environmentally sustainable food, prevent promotion of unhealthy and unsustainable food, improve skills and knowledge related to healthy and environmentally sustainable food, and reduce the environmental impact of food sold and consumed on campus. The ‘food retail outlet’ component assesses food retail outlets in relation to the availability, accessibility, promotion, price, and likely environmental impact of food sold, as well as the information provided at point of sale.

### 3.2. Process for Assessment of University Food Environments Using the Uni-Food Tool

**Figure 1:**
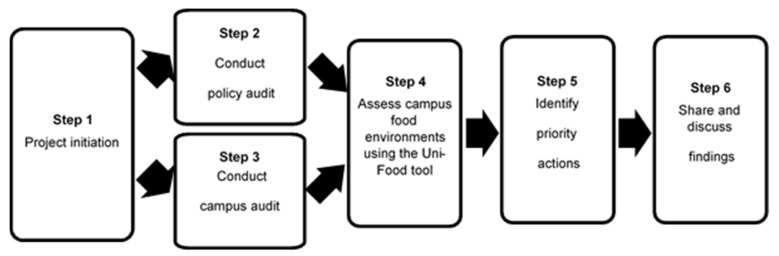
Process for implementing the University Food Environment Assessment (Uni-Food) tool.

The process for implementation of the Uni-Food tool is depicted in Figure 1 and described in detail below. 

#### 3.2.1. Step 1: Project Initiation

Universities wishing to self-assess their food environments using the Uni-Food tool would need to initiate the project by establishing a project team, typically including: a senior staff member to oversee the project, members within campus facilities management and services with knowledge of relevant university policies and facilities, and a small number of assessors (the number will vary based on the number of campuses and food retail outlets to be assessed). Organisational consent (e.g., from a senior university official) may also need to be obtained to facilitate the project.

The tool would then need to be tailored to the particular university context. For example, adjustments to the assessment criteria may be necessary to account for relevant state- or local-level healthy food provision guidelines, and indicators that are not applicable should be removed (e.g., if the university does not provide student accommodation, then related indicators are not required). The campuses and food retail outlets to be included in the scope of the assessment also need to be determined. If multiple campuses and food retail outlets are to be assessed, the project team may decide to weight the assessment of particular campuses and food retail outlets to reflect their relative importance, based on population served, prominence and/or revenue. The most suitable time of year to conduct the assessment also needs to be determined. Ideally, the assessment should be conducted when the campus is most active.

Training of the assessors in the use of the Uni-Food tool (including scope, related definitions, and process of assessment) is conducted by an external research team member familiar with the Uni-Food tool (e.g., as part of the INFORMAS network). There is no expectation that assessors have prior knowledge of the topic areas to be assessed as part of the tool, and the role would suit students currently enrolled at the university being assessed.

#### 3.2.2. Step 2: Conduct Policy Audit

In the policy audit, assessors collect evidence of relevant university policies and identified in the ‘university systems and governance’ component of the tool. Evidence can be found in strategy documents, policies, and procedures. Relevant documents are typically found on the university website, intranet, and shared storage. Where required, further input may be needed from university staff members with knowledge of policies in development or processes not formally documented or available to students and/or the general public. 

#### 3.2.3. Step 3: Conduct Campus Audit

On campus data collection involves an audit of relevant campus environments and food retail outlets using a custom-developed audit tool (available on request). It is recommended that data collection is carried out independently by at least two assessors based at the university, with supervision from a university senior staff member. 

The campus audit requires visual inspection of water taps/fountains, waste management facilities, vending machines, and fundraising and promotional activities at the university. Multiple campus visits may be required to assess changes in practices at different times of the year.

For each food retail outlet, the audit requires visual inspection of food and beverage displays and menus (if available), nutritional and food labelling, waste monitoring practices, serving ware use, and advertisement and promotional activities within the outlet. It is recommended that assessors notify the retail outlet of the purpose of the audit before collecting relevant data. A detailed analysis of the nutritional composition of the foods and beverages available at each outlet is not required, but, if resources permit, could be used to supplement the Uni-Food assessment. Liaison with the food retail outlet staff members and manager may be required to understand each food retail outlet’s environmental sustainability policies and practices. Due to variability in campus retailers’ opening hours, for some campuses, more than one campus audit may need to be conducted to capture retailer regular practice. 

#### 3.2.4. Step 4: Assess Campus Food Environments Using the Uni-Food Tool

Data collected in Steps 2 and 3 are collated by the assessors. Scores are assigned to each indicator based on the assessment criteria (as outlined in Table S3). An overall score for each of the three components of the tool and the university’s food environment overall (expressed as a score out of 100) are calculated based on the generic and university-specific weightings (see Table S2).

#### 3.2.5. Step 5: Identify Priority Actions

The project team then develops university-specific recommendations to improve campus food environments based on the assessment results, feasibility considerations and available good practice examples for each domain. The university project team are encouraged to liaise with university stakeholders to tailor recommendations to their institutional context, strategic planning, and operational requirements.

#### 3.2.6. Step 6: Share and Discuss Findings 

It is recommended that results for each university are collated into a report that provides a summary of the assessment process, a scorecard of the results, and recommended actions. This step of the process can also be used to identify areas where support (e.g., expertise, resources) is likely to be needed to progress recommended actions.

### 3.3. Uni-Food Tool Pilot Implementation

**Figure 2:**
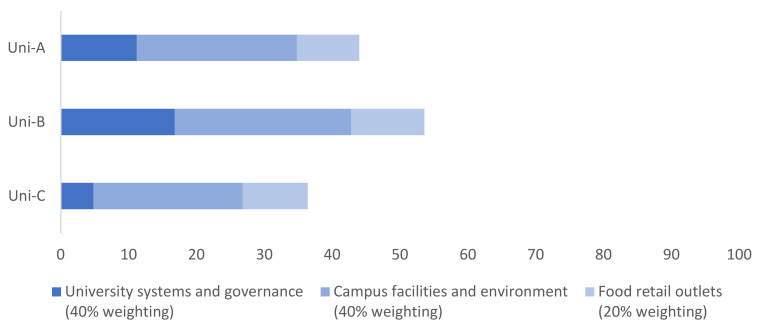
Pilot assessment (score out of 100) of the healthiness, equity, and environmental sustainability of food environments at three Australian universities using the University Food Environment Assessment (Uni-Food) tool.

In the pilot implementation of the Uni-Food tool, the overall scores for the selected universities ranged from 34% to 54% (Uni-A = 44%, Uni-B = 54%, Uni-C = 36%) (Figure 2 and Table 2). The ‘university systems and governance’ component had the lowest scores and the most variation, with scores ranging from 7% to 42% (Uni-A = 28%, Uni-B = 42%, Uni-C = 12%). The ‘campus facilities and environment’ component was the highest scoring component for each university (Uni-A = 59%, Uni-B = 65%, Uni-C = 55%), followed by the ’food retail outlets’ component (Uni-A = 46%, Uni-B = 54%, Uni-C = 48%). ‘Policies for food retail environments’ (under the ‘university systems and governance’ component) was the lowest scoring domain, with all universities receiving 5% in this domain. The domains in which universities scored consistently well were ‘personal and community development’ (Uni-A = 78%, Uni-B = 72%, Uni-C = 72%), and ‘environmental impact’ (Uni-A = 76%, Uni-B = 83%, Uni-C = 73%), both within the ‘campus facilities and environment’ component. All universities scored moderately well in the ‘promotion’ domain (Uni-A = 69%, Uni-B = 81%, Uni-C = 65%) within the ‘food retail outlets’ component.

Key strengths identified across all universities included their strong sustainable processes relating to energy and water use, and their representation of students on key working groups related to the promotion of healthy, equitable and environmentally sustainable campus food environments. Key areas where universities were found to fall short of best practice recommendations included: a lack of policies to increase the availability and affordability of healthy and environmentally sustainable food options on campus; a lack of display of nutrition and environmental impact information at point of sale in food retail outlets; a lack of policies prohibiting the sale and promotion of unhealthy and unsustainable products (e.g., ban on sugar-sweetened beverages). 

Inter-rater reliability of the scoring of universities using Gwet’s AC1 (unweighted) integrated reliability coefficients were similar between universities (Uni-A: 0.74 (95% confidence interval [CI]: 0.64–0.78; Uni-B: 0.73 (95% CI: 0.65–0.76); Uni-C: 0.70 (95% CI: 0.61–0.73).

University engagement with the process was high as evidenced by the high completion rate of the audit (data was collected for all indicators, in all three pilot universities) and the willingness of the project team at each pilot university to complete future assessments of their food environments using the Uni-Food tool. The findings from the evaluation survey (refer to upplementary File S1, Supplementary File S2 and Table S4) indicated that assessors found the tool easy to use, the training useful, and believed it to be an effective tool to assess university food environments. No financial cost was incurred by any of the universities to complete the audit. The median time required to complete the process was 45 h (inter quartile range: 37.5–80.0 h) for assessors and <10 h by supervisors over approximately four weeks to guide the assessors to complete the audit.

## 4. Discussion

This paper described the development of a tool and process to benchmark the healthiness, equity, and environmental sustainability of food environments in tertiary education settings, and its pilot implementation in 3 Australian universities in 2021. The Uni-Food tool consists of 3 components, 68 indicators, across 16 domains, informed by a review of the literature and input from an expert advisory panel. The comprehensive tool is tailored to the university setting, with a reliable self-implementation process for universities that includes engagement with key stakeholders, requires only minimal resources, and is suitable for wide-scale implementation across the tertiary education sector.

Our pilot application of the tool in a small sample of Australian universities indicated that current Australian university environments are not meeting best practice guidelines for creating healthy, equitable and environmentally sustainable food environments as evidenced by the low overall scores obtained (36–54%). The results highlighted that, amongst the included universities, there was some diversity in the healthiness and environmental sustainability of their food environments, but they all lacked comprehensive policies and strategies related to food environments. In particular, we found an almost complete lack of strategies and commitments related to the availability, labelling, accessibility, and affordability of healthy and environmentally sustainable foods provided in vending machines and within food retail outlets on campus. We also found an absence of policies to restrict the availability and promotion of unhealthy and unsustainable foods and beverages.

The assessment results from this study are consistent with the literature demonstrating that university food environments lack an availability of healthy foods and promote the consumption of ultra-processed discretionary foods and beverages [29,32,40]. In particular, recent evaluations of campus food environments in Brazil, New Zealand, Norway and Canada suggest they generally do not support healthy eating [30,32,33,40,41]. Within Australia, an assessment of the healthiness of foods provided within food retail outlets across seven universities showed that the majority of food products were classified as unhealthy [29]. 

Encouragingly, the results from this pilot study suggest that universities within Australia are taking substantial steps in addressing and managing the environmental impact of campus facilities and services. However, these efforts need to be expanded to specifically focus on the environmental impact of food systems and food environments. This is consistent with several studies that have explored actions taken by universities to promote environmentally sustainable food practices [22,42,43]. While there have been some positive steps taken in the U.S.A in particular, the implementation of initiatives has been piecemeal with few examples of universities that have adopted a comprehensive suite of actions [44,45].

Universities are a promising setting for advancing food environment transformation, particularly considering the potential role that the tertiary education sector can play in leading innovation and social change [46]. Recently, universities have shown leadership in advancing social and institutional change [46,47]. In particular, attention to sustainable development in the tertiary education sector has flourished following the UNESCO *Decade of Education for Sustainable Development* and the uptake of a number of international declarations committing universities to take action on environmental sustainability through research, curriculum and campus operations [48]. This has been supported by the development of global networks for Education for Sustainable Development to share progress and best practice between institutions [49]. The Sustainable Development Goals have become a strong organising principle for action in this area, with the Higher Education Sustainability Initiative (HESI) acting as a conduit between Higher Education Institutions, Policy Makers and the United Nations [50]. 

Benchmarking and ranking universities on a range of performance indicators is a common practice that has accelerated in recent years [51]. Due to the competitive nature of the tertiary education sector, benchmarking activities have proved powerful in catalysing policy and governance change at the institutional level [51]. For example, the sector’s sustainable development efforts have been enhanced in response to the development and broad uptake of initiatives such as the STARS accreditation scheme [28,48]. Widespread application of the Uni-Food tool and its repeated use over time will garner a more comprehensive understanding of the current state of university food environments, increase awareness of best practice, and enable more detailed benchmarking. This will facilitate a direct comparison between universities and is anticipated to be an effective tool in driving policy and practice change. Wide-spread application of similar benchmarking efforts such as the Healthy Food Environment Policy Index (Food-EPI) and the BIA-Obesity initiative within Australia and globally have shown to be effective in advocating for and helping to provoke policy and company change and holding companies and governments accountable for their role in improving food environments [14,37,52]. 

The pilot application of the Uni-Food tool resulted in a range (36–54%) in the overall scores received for each university, suggesting that the Uni-Food tool is sensitive enough to discriminate between different university food environments. Good inter-rater reliability of the tool places confidence in the ability of the tool to provide consistent scores, based on detailed and comprehensive evidence. However, we note that further reliability testing should be conducted with assessors who are not familiar with the tool [53]. Overall, the findings from the evaluation of the pilot indicate that the tool was considered easy to use and useful in assessing the university food environment. Furthermore, the relatively small time commitment and minimal financial cost to apply the tool suggests that regular benchmarking of university food environments would be plausible. Multiple assessments over time would allow universities to monitor and track progress over time, provide a mechanism to increase accountability, and provide a framework to guide best practice action. As the tool is applied more broadly, it will be important to measure the effectiveness and cost-effectiveness of the Uni-Food tool in assisting in the implementation of recommended policy and practice change.

While the Uni-Food tool has been developed for the Australian context, it is envisaged that the tool can readily be adapted for application to other countries. This will require the tool to be tailored to the specific context in which it will be used, consistent with the way in which other tools developed by INFORMAS (e.g., Food-EPI and BIA-Obesity) are applied [13,37]. Country-level adaptation of the tool is likely to maximise the relevance of the results within a particular country but may limit the extent to which universities can be compared across countries. Nevertheless, an international application of the tool will help to further develop examples of international best practice. It is also anticipated that the Uni-Food tool can be adapted for assessment of other settings with complex food environments, such as food courts, shopping centres and local activity areas (e.g., “high streets”). Although components and domains are likely to remain similar when applied to other settings, it is expected that indicator and related assessment criteria will need to be modified to suit the characteristics of the setting and the regulatory context. It is imperative that any adapted tools be pilot tested and evaluated before widespread application. Further iterations of the tool to enable such assessments could be explored as part of the efforts of INFORMAS to monitor and benchmark food environments globally.

### Strengths and Limitations of the Uni-Food Tool

The development of the Uni-Food tool and process was an iterative approach based on the best-available evidence. The indicators and criteria for assessment were developed based on an extensive review of the literature, and thorough consultation with an expert advisory panel. A key strength of the Uni-Food tool is that the indicators incorporate aspects of health, equity, and environmental sustainability. The inclusion of equity and environmental sustainability into the tool was in recognition of the important role that food environments play in influencing equitable access to food, and the substantial impact of food systems on environmental outcomes. This is a highly novel contribution to the food environment literature as the vast majority of tools developed to date have only focused on health-related aspects of food environments. Equity and environmental sustainability considerations should be considered as part of future iterations of other tools to monitor and benchmark different components of food environments.

The Uni-Food tool and process has some limitations. Firstly, the design of the tool does not include a detailed analysis of the nutritional composition or environmental impact of food and beverage products available. We note that several detailed audit tools already exist for application at the food retail outlet level to assess the nutritional composition of foods (e.g., Nutrition Environment Measures Surveys [41]) that could be used in conjunction with Uni-Food to provide this detail if needed and if resources allow. However, currently, no detailed audit tool exists to assess the environmental impact of different foods, which is most likely due to the complexity in the calculation of the environmental impact of specific food products. Existing institutional guidelines (e.g., for food provision and retail) regarding the environmental sustainability of different foods in the Australian context are mostly qualitative, and environmental sustainability is not incorporated into the Australian Dietary Guidelines in a comprehensive way [34,36]. As such, a broad definition of healthy, equitable and environmentally sustainable foods was employed as part of the Uni-Food tool. Whilst the way in which environmental sustainability was operationalised as part of the tool provides high-level guidance on ways to incorporate environmental sustainability considerations, and is helpful in highlighting the interconnected nature of health and environmentally sustainability, more detailed operational definitions and standardised measures are likely to be needed to provide specific guidance at the food product level and for gauging progress [47]. A further limitation of the Uni-Food tool is that the scope did not include other aspects of health, such as physical activity, alcohol, and tobacco, which are heavily influenced by environmental factors [54]. As such, universities wishing to improve the healthiness of campus environments in a comprehensive way would benefit from supplementing the Uni-Food tool with additional assessments and related policy measures. The Victorian Government’s Achievement program, that assesses institutions on a broad range of health measures, provides some guidance in this area [55].

## 5. Conclusions

Universities can play an important role in driving food environment transformation to enhance population and planetary health. The results from this study suggest that there is currently a substantial opportunity for Australian universities to improve their food environments. Such actions can positively influence the dietary patterns of students and staff, and simultaneously demonstrate leadership in advancing social and institutional change. The development of the Uni-Food tool and process provides a comprehensive and reliable mechanism to benchmark university food environments regarding their healthiness, equity, and environmental sustainability which was lacking in previous benchmarking and monitoring tools. Widespread implementation of the tool can be used to increase accountability for action and build awareness of best practice. The impact of the tool in contributing to policy and practice change, and its overall influence on the healthiness, equity and environmental sustainability of university food environments remains to be evaluated.

## Figures and Tables

**Table 1:** Overview of components, domains and sub-domains of the University Food Environment Assessment (Uni-Food) tool.

Component	Domain	Sub-Domain (Number of Indicators, *n*) ^1^
University systems and governance	Leadership and planning	Policies and commitments (*n* = 2)
	Policies for food retail environments	Proportion of healthy and environmentally sustainable food and beverages sold (*n* = 1)
		Restrictions on availability (*n* = 1)
		Food pricing (*n* = 1)
		Labelling and information (*n* = 2)
		Food retail contracts (*n* = 1)
	Monitoring and reporting	Food environments (*n* = 1)
		Staff and student population (*n* = 2)
	Funding and resources	Funding (*n* = 1)
		Resources (*n* = 2)
	Stakeholder engagement	Platforms for interaction (*n* = 1)
		Student voice (*n* = 3)
Campus facilities and environment	Availability and accessibility	Drinking water (*n* = 1)
		Healthy, equitable and environmentally sustainable food (*n* = 1)
		Culturally appropriate food (*n* = 2)
		Vending machines (*n* = 3)
		Self-catering facilities (*n* = 1)
		Operating hours (*n* = 1)
	Equity	Food affordability (*n* = 2)
		Food relief (*n* = 1)
	Advertising and sponsorship	Advertising (*n* = 1)
		Sponsorship (*n* = 1)
	Catering and events	Catering (*n* = 1)
		Fundraising (*n* = 1)
		Student accommodation (*n* = 1)
	Personal and community development	Community skills building (*n* = 3)
		Training and information (*n* = 3)
	Environmental impact	Waste and recycling (*n* = 3)
		Food packaging and serving ware (*n* = 1)
		Water (*n* = 1)
		Energy and emissions (*n* = 1)
Food retail outlets	Availability and accessibility	Healthy, equitable and environmentally sustainable foods and beverages (*n* = 2)
		Portion sizes (*n* = 1)
		Location of foods (*n* = 1)
	Promotion	Food and beverage advertising (*n* = 1)
	Price	Relative prices (*n* = 2)
		Price promotions (*n* = 2)
	Information	Nutrition information (*n* = 1)
		Cultural information (*n* = 1)
		Environmental sustainability information (*n* = 1)
	Environmental impact	Food packaging and serving ware (*n* = 2)
		Food waste (*n* = 1)

Table notes: ^1^ Indicators and assessment criteria for each sub-domain are provided in Table S3.

**Table 2:** Results from pilot assessment of the healthiness, equity, and environmental sustainability of food environments at three Australian universities using the University Food Environment Assessment (Uni-Food) tool.

		Uni-A	Uni-B	Uni-C
Number of Food Retailers on Main Campus Assessed		10	21	17
Overall university score ^1^		44%	54%	36%
Component (unweighted) ^2^	Domain (unweighted) ^3^			
University systems and governance ^2^		28%	42%	12%
	Leadership and planning ^3^	63%	50%	25%
	Policies for food retail environments ^3^	5%	5%	15%
	Monitoring and reporting ^3^	42%	67%	0%
	Funding and resources ^3^	58%	100%	8%
	Stakeholder engagement ^3^	40%	65%	5%
Campus facilities and environment ^2^		59%	65%	55%
	Availability and accessibility ^3^	54%	67%	63%
	Equity ^3^	48%	60%	67%
	Advertising and sponsorship ^3^	75%	50%	0%
	Events and catering ^3^	38%	58%	25%
	Personal and community development ^3^	78%	72%	72%
	Environmental impact ^3^	76%	83%	73%
Food retail outlets ^2^		46%	54%	48%
	Availability and accessibility ^3^	44%	49%	52%
	Promotion ^3^	69%	81%	65%
	Price ^3^	50%	60%	49%
	Information ^3^	20%	31%	26%
	Environmental impact ^3^	39%	49%	46%

Table notes: ^1^ Weighted scores shown, including weighting at the component, domain and sub-domain levels. Refer to Table S2 for weightings. No university-specific weightings applied. ^2^ Scores shown include weighting at the domain and sub-domain level only i.e., weighting at the component level not applied. Refer to Table S2 for weightings. No university-specific weightings applied. ^3^ Scores shown include weighting at the sub-domain level only i.e., weighting at the domain level not applied. Refer to Table S2 for weightings. No university-specific weightings applied.

## Data Availability

The data presented in this study are available on request from the corresponding author. The data are not publicly available due to privacy issues.

## Supplementary Materials

The following are available online at www.mdpi.com/article/10.3390/ijerph182211895/s1. Table S1: Relevant existing tools benchmarking and assessing food environments that informed the development of the Uni-Food tool and process, Table S2: Uni-Food tool component, domain and sub-domain, Table S3: Uni-Food tool subdomains, indicators, and associated assessment criteria, File S1: Questionnaire assessor evaluation, File S2: Results assessor evaluation, Table S4: Assessors’ (n = 6) evaluation of the Uni-Food tool and training session. References [7,29,34,37,41,55,56,57,58,59,60,61,62,63,64] are cited in the supplementary materials.
