# Supplementary material for: Development of the University Food Environment Assessment (Uni-Food) Tool and Process to Benchmark the Healthiness, Equity, and Environmental Sustainability of University Food Environments
Source: Int J Environ Res Public Health. 2021 Nov 12;18(22):11895. doi: 10.3390/ijerph182211895 (PMC8625487; doi:10.3390/ijerph182211895)
Supplement: Supplementary file 1 [file ijerph-18-11895-s001.zip › ijerph-1384653-supplementary.pdf]

International Journal of
Environmental Research and Public Health

MDPI

Development of the University Food Environment Assessment (Uni-Food) tool and process to benchmark the healthiness, equity, and environmental sustainability of university food environments

Online Supplementary material:

Table S1. Relevant existing tools benchmarking and assessing food environments that informed the development of the Uni-Food tool and process.

Name	Focus Area	Setting	Key Areas of Measurement Relevant to University Food Environments
Fair Food Challenge (1)	Environmental sustainability, equity and health	Tertiary education institutions	Environmental sustainability; access and equity; health; education; innovation and opportunity; procurement and retail; resourcing and accountability
Food Environment Quality Index (2)	Nutrition	Food outlets on tertiary education campuses	Availability; accessibility; promotion
Healthy Food Environment Policy Index (Food-EPI) (3)	Nutrition	National and state government	Infrastructure support: Leadership; governance; monitoring and intelligence; funding and resources; platforms for interaction; workforce development; health-in-all policies Policies: food composition; food labelling; food promotion; food provision; food retail; food prices; food trade and investment
Healthy Universities (4)	Health and wellbeing	Tertiary education institutions	Leadership and governance; service provision; facilities and environment; communication; information and marketing; academic; personal, social and professional development
Menus of Change (5)	Human and planetary health	Food service industry	Transparent procurement; seasonal, local, and minimally processed food; sustainable and ethical practices; plant-forward; reduced portions
Nutrition Environment Measures Study in Restaurants (NEMS-R) (6)	Nutrition	Restaurants	Availability; facilitators of healthy eating; barriers to healthful eating; pricing; signage
Nutrition Environment Measures Study (NEMS) Grab-and-go (7)	Nutrition	Food outlets on tertiary education campuses	Signage; availability; price
Nutrition Environment Measures Study University Campuses (NEMS-UC) (8)	Nutrition	Food outlets on tertiary education campuses	Availability of healthy options; facilitators to healthy eating; barriers to healthy eating; promotions; food-beverage placement
Policies, Opportunities, Initiatives and Notable Topics (POINTS) (9)	Health and wellbeing	Tertiary education institutions	Healthy food options; nutrient standards; healthy food labels and point-of-purchase nutrition info; food taxes and subsidies; designated eating environments; local and sus-

			tainable food; organic waste reduction and disposal; farmers markets; local food access on campus; campus food gardens
STARS (10)	Environmental sustainability	Tertiary education institutions	Food and beverage purchasing; sustainable dining; waste; planning and administration; engagement; academics
University Nutrition Environment Assessment Tool (U-NEAT) and University Nutrition Program Assessment Tool (U-NPAT) (11) [1]	Nutrition	Tertiary education institutions	U-NEAT: Food availability; pricing; food service practices; nutrition standards and policies; provision of nutrition information<br>U-NPAT: Nutrition assessments and non-academic nutrition education; health centre staffing; nutrition policies

[1] Provided to the research team via communication with the authors

Table S2. Uni-Food tool component, domain and sub-domain weightings.

COMPONENT	WEIGHTING [1]	DOMAIN	WEIGHTING [2]	SUB-DOMAIN	WEIGHTING [3]
University systems and governance	40%	Leadership and planning	10%	Policies and commitments	100%
		Policies for food retail environments	50%	Proportion of healthy and environmentally sustainable food and beverages sold	20%
				Restrictions on availability	20%
				Food pricing	20%
				Labelling and information	20%
				Food retail contracts	20%
		Monitoring and reporting	10%	Food environments	50%
				Staff and student population	50%
		Funding and resources	20%	Funding	50%
				Resources	50%
		Stakeholder engagement	10%	Platforms for interaction	50%
				Student voice	50%
Campus facilities and environment	40% [4]	Availability and accessibility	20%	Drinking water	13%
				Healthy, equitable and environmentally sustainable food	13%
				Culturally appropriate food	13%
				Vending machines	40%

		Self-catering facilities	13%
		Operating hours	7%
Equity	25%	Food Affordability	66%
		Food relief	33%
Advertising and sponsorship	10%	Advertising	50%
		Sponsorship	50%
Catering and events	15%	Catering	33%
		Fundraising	33%
		Student accommodation	33%
Personal and community development	15%	Community skills building	88%
		Training and information	22%
Environmental impact	15%	Waste and recycling	56%
		Food packaging and serving ware	22%
		Water	11%

				Energy and Emissions	11%
Food retail environment	20% [5]	Availability and accessibility	25%	Healthy, equitable and environmentally sustainable foods and beverages	50%
				Portion sizes	25%
				Location of foods	25%
		Promotion	15%	Food and beverage advertising	100%
		Price	25%	Relative price	57%
				Price promotions	43%
		Information	15%	Nutrition information	30%
				Cultural information	30%
				Environmental sustainability information	30%
		Environmental impact	20%	Food packaging and serving ware	66%
				Food waste	33%

[1] Weighting contribution of each component as part of the total score; [2] Weighting contribution of each domain as part of the score for that component; [3] Weighting contribution of each sub-domain as part of the score for that domain; [4] Where universities have multiple campuses, each campus can be allocated a separate score. The contribution of each campus score to the overall components score can be adjusted by allocation of a relative weighting for each campus based on the size of campus and proportion of staff and students at each campus; [5] Assessment is conducted for every food retailer on the university campus and provided a separate score. The contribution of each food retailer sore to the components overall score can be adjusted by allocation of a relative weighting for each food retail outlet based on their size, proportion of staff and students that they serve and/or their relative influence on the university food environment.

Table S3. Uni-Food tool subdomains, indicators, and associated assessment criteria.

Sub Domain	Indicator	Assessment Criteria
**UNIVERSITY SYSTEMS AND GOVERNANCE**		
**LEADERSHIP AND PLANNING (LSHIP)**		
**LSHIP1**	Does the university have a detailed plan or strategy to promote healthy, equitable and environmentally sustainable campus food environments?	10: There is a publicly available, comprehensive and up-to-date plan or commitment outlining measurable and time-bound objectives and strategies to promote healthy, equitable and environmentally sustainable food environments.<br>7.5: There is a publicly available plan or commitment outlining objectives and strategies to promote healthy, equitable and environmentally sustainable food environments, but it is either not comprehensive in the areas covered, or the measures are not specific, measurable or time-bound.<br>5: There is a publicly available plan or commitment outlining objectives and strategies to promote the healthiness, equity or environmental sustainability of campuses, but it is not specifically related to food.<br>2.5: No current plan or commitment but work underway to develop a comprehensive plan outlining objectives and strategies to promote healthy, equitable and environmentally sustainable environments<br>0: No evidence/No plan or commitment outlining objectives or strategies to promote healthy, equitable and environmentally sustainable campus environments
**LSHIP2**	Which aspects of healthy, equitable and environmentally sustainable campus food environments are included in university plans and policies?	Award 2.5 points for each of the following aspects included in university plans or policies:<br>- Healthiness of campus food<br>- Environmental sustainability of campus food<br>- Cultural diversity of campus food<br>- Affordability of campus food

POLICIES FOR FOOD RETAIL ENVIRONMENTS (POL)		
**POL1**	Does the university have a policy to increase the number/proportion of healthy and environmentally sustainable food and beverages * sold on campus?	10: There are university-wide targets in place regarding the proportion of healthy and environmentally sustainable products sold in campus food retail outlets and vending machines, with regular reporting on progress<br>5: General commitment to implementing a policy to increase the proportion of healthy and environmentally sustainable products sold in campus food retail outlets and vending machines/targets in place in this area, but no regular reporting on progress/targets only apply in limited settings<br>2.5: Development of a policy/targets to increase the proportion of healthy and environmentally sustainable products sold in campus food retail outlets and vending machines underway<br>0: No policy/commitment/strategy exists
**POL2**	Does the university have a policy in place to restrict availability of certain categories of unhealthy/environmentally unsustainable products* (e.g. sugary drinks)?	10: There is a university-wide policy in place to ban the sales of certain categories of unhealthy/environmentally unsustainable products (e.g. sugary drinks) on campus<br>5: General commitment to reduce/phase out the sales of certain categories of unhealthy/environmentally unsustainable products (e.g. sugary drinks) on campus<br>2.5: Policy under development<br>0: No policy
**POL3**	Does the university have a policy to ensure that a range of healthy and environmentally sustainable foods and beverages* are affordably priced and their purchase/consumption is incentivised relative to unhealthy products?	10. There is a policy in place to ensure that a range of healthy and environmentally sustainable foods and beverages (including snacks and ‘main meals’) available for purchase on campus are affordably priced and their purchase/consumption is incentivised relative to unhealthy products (including products available in nearby retailers)<br>5: General stated commitment to address affordability and pricing of healthy and environmentally sustainable foods and beverages/ongoing implementation of a targeted policy in this area<br>2.5: Development of a policy around affordability and pricing of healthy and environmentally sustainable foods and beverages underway<br>0: No policy/commitment/strategy exists

**POL4**	Does the university have a policy that food retail outlets and vendors provide nutrition information for food and beverages at point of sale?	10: There is a fully implemented policy (applied and actively monitored across all/most outlets) stipulating that all vendors/food retail outlets display easy-to-understand nutrition labelling at point of sale for all products, with classification based on national or state classification systems<br>5: There is a policy stipulating that vendors/food retail outlets display easy-to-understand nutrition labelling at point of sale, but this is only partially implemented in practice (eg. doesn't apply to all food retail outlets, or not to all products)<br>2.5: No policy exists but the university encourages food retailers to display nutrition and/or environmental sustainability information/policy development underway<br>0: No policy exists
**POL5**	Does the university have a policy that food retail outlets and vendors provide environmental sustainability information for food and beverages at point of sale?	This indicator is currently not being assessed
**POL6**	Does the university incorporate commitments to healthy, equitable and environmentally sustainable food* into retail and procurement contracts/agreements?	10: All food retail and procurement contracts/agreements include comprehensive commitments to healthy, equitable and environmentally sustainable food.<br>7.5: All new and renewing food retail and procurement contracts/agreements include comprehensive commitments to healthy, equitable and environmentally sustainable food.<br>5: Some or all food retail and procurement contracts/agreements include commitments to healthy, equitable and/or environmentally sustainable food, however the extent of commitments are not extensive.<br>2.5: Work is underway to include commitments to healthy, equitable and/or environmentally sustainable food in food retail and procurement contracts/agreements.<br>0: No evidence/ No commitment to healthy, equitable and/or environmentally sustainable food in food retail and procurement contracts/agreements.

	MONITORING AND REPORTING (MONTR)	
**MONTR1**	Does the university regularly measure the healthiness, sustainability and environmental sustainability of campus food environments?	10: A comprehensive array of indicators related to the healthiness, equity and sustainability of campus food environments are regularly measured and publicly reported<br>7.5: A comprehensive array of indicators related to the healthiness, equity and sustainability of campus food environments are measured, but this is infrequent and/or not publicly reported<br>5: A limited number of indicators related to the healthiness, equity and sustainability of campus food environment are measured<br>2.5: Planning is underway to measure the healthiness, equity and sustainability of campus food environments<br>0: No evidence/ No measurement of indicators related to the healthiness, equity or environmental sustainability of campus food environments
**MONTR2**	Does the university regularly survey staff and students about dietary needs and preferences?	10: A comprehensive survey of staff and student dietary needs and preferences is regularly conducted<br>7.5: A comprehensive survey of staff and student dietary needs and preferences is irregularly conducted<br>5: Some aspects of staff and student dietary needs and preferences are surveyed<br>2.5: Planning or discussions are underway regarding a survey of staff and student dietary needs and preferences<br>0: No evidence/ No survey of staff and student dietary needs or preferences
**MONTR3**	Does the university regularly measure staff and student food-related health outcomes?	10: A comprehensive array of staff and student food-related health outcomes are regularly measured<br>7.5: A comprehensive array of staff and student food-related health outcomes are irregularly measured<br>5: A limited number of staff and student food-related health outcomes are measured<br>2.5: Planning or discussions are underway regarding measurement of staff and student food-related health outcomes<br>0: No evidence/ No measurement of staff and student food-related health outcomes.

FUNDING AND RESOURCES (RESOUR)		
**RESOUR1**	Does the university allocate funding for initiatives to promote healthy, equitable and environmentally sustainable campus food environments?	10: Dedicated and recurrent funding is allocated to support initiatives for healthy, equitable and environmentally sustainable campus food environments<br>5: Funding is allocated to support initiatives for healthy, equitable and environmentally sustainable campus food environments, however the funding allocation is ad-hoc<br>0: No evidence/ No clear, dedicated funding to support initiatives for healthy, equitable and environmentally sustainable campus food environments
**RESOUR2**	Does the university employ staff to conduct work to improve the healthiness, equity and environmental sustainability of campus food environments?	10: There are staff dedicated to support the development, implementation and monitoring of initiatives related to healthy, equitable and environmentally sustainable campus food environments<br>5: There are staff that conduct some work related to healthy, equitable and environmentally sustainable food environments on campus but it is not their core work function<br>0: No evidence/ No dedicated staff to work on initiatives related to healthy, equitable and environmentally sustainable campus food environments
**RESOUR3**	Is there a cross-university group to coordinate action on campus food environments?	10: There is a cross-university group with representation from a diverse range of work functions that have an active and ongoing program of work to support policies and initiatives to promote healthy, equitable and environmentally sustainable campus food environments<br>7.5: There is a cross-university group with representation from a diverse range of work functions to support policies and initiatives to promote healthy, equitable and environmentally sustainable campus food environments, but the work program is ad-hoc in nature<br>5: There is a cross-university group, but diversity of membership is very limited and work program is ad-hoc in nature<br>2.5: No current cross-university working group, but efforts underway to convene a group<br>0: No evidence/ No cross-university group in place to discuss policies and initiatives to promote healthy, equitable and environmentally sustainable campus food environments

STAKEHOLDER ENGAGEMENT (ENGAGE)		
**ENGAGE1**	Does the university ensure staff and students contribute to the vision and design of campus food environments?	10: Staff and student input is regularly invited and promoted when developing new strategies or activities to promote healthy, equitable and environmentally sustainable campus food environments.<br>7.5: Staff and student input is occasionally invited when developing new strategies or activities to promote healthy, equitable and environmentally sustainable campus food environments.<br>5: Staff and student input is invited when developing new strategies or activities to promote healthy, equitable and environmentally sustainable campus food environments, but involvement is limited or late in the development process, thereby reducing meaningful input.<br>2.5: Work is underway to gather staff and student input into the development of strategies or activities to promote healthy, equitable and environmentally sustainable campus food environments.<br>0: No evidence/ No staff or student input sought when developing new strategies or activities to promote healthy, equitable and environmentally sustainable campus food environments
**ENGAGE2**	Are students represented on key working groups responsible for promoting healthy, equitable and environmentally sustainable campus food environments?	10: Multiple students that reflect the diversity of the student population are represented on key working groups / committees related to healthy, equitable and environmentally sustainable campus food<br>5: There is student representation on key working groups / committees related to healthy, equitable and environmentally sustainable campus food, but involvement is limited in either frequency OR diversity<br>2.5: There is student representation on key working groups / committees related to healthy, equitable and environmentally sustainable campus food, but involvement is limited in both frequency AND diversity<br>0: No evidence/ Students are not represented on key working groups / committees related to healthy, equitable and environmentally sustainable campus food<br>N/A: The university does not have any working groups/ committees related to healthy, equitable or environmentally sustainable campus food
**ENGAGE3**	Are there systems in place that promote communication and partnership between the university and student	10: There is a formal partnership or regular meetings between the university and key student organisations to facilitate discussions regarding the healthiness, equity and environmental sustainability of campus food

	organisations related to campus food environments?	5: Informal and irregular discussions between the university and key student organisations regarding the healthiness, equity and environmental sustainability of campus food<br>0: No clear communication between the university and key student organisations regarding the healthiness, equity and environmental sustainability of campus food
**ENGAGE4**	Are there systems in place that promote communication and partnership between the university and external stakeholders relevant to campus food environments?	10: There is a formal partnership or regular meetings between the university and relevant external organisations to facilitate discussions regarding the healthiness, equity and environmental sustainability of campus food<br>5: Informal and irregular discussions with relevant external stakeholders regarding improving the healthiness, equity or environmental sustainability of campus food environments<br>0: No evidence/no observable systems in place to promote partnership with relevant external stakeholders on the healthiness, equity or environmental sustainability of campus food environments
**CAMPUS FACILITIES AND ENVIRONMENT**		
**AVAILABILITY AND ACCESSIBILITY (AVAIL)**		
**AVAIL1**	Is free drinking water provided and readily accessible across campus?	10: Free drinking water is widely available across all campuses<br>7.5: Free drinking water is moderately available across campuses<br>2.5: There is limited free drinking water across campuses<br>0: Free drinking water is not available on campus
**AVAIL2**	Is there a store or market on campus where fresh produce and minimally processed grocery items can be purchased?	10: There is a store or stall on or within 500m of all campuses where fresh produce and minimally processed items can be purchased.<br>7.5: There is a store or stall on or within 500m of all campuses where a fresh produce and minimally processed items can be purchased, but it is not open every day (e.g., weekly market pop-up stall).<br>5: There is a store or stall on or within 500m of some campuses where fresh produce and minimally processed items can be purchased.<br>2.5: There is a store where fresh produce and minimally processed items can be purchased but the selection of produce is very limited (e.g. small select of fruit available in a cafe/convenience store).<br>0: There is no store or stall on or within 500m of campuses where fresh produce and minimally processed grocery items can be purchased.

		Tip: Stores or stalls may include supermarkets, convenience store with a dedicated fresh produce section, food co-ops, farmers market stalls.
**AVAIL3**	Is a diverse range of culturally appropriate foods available across campus?	10: A variety of foods and/or beverages from different cuisines and dietary requirements are widely available for purchase across all areas of the university<br>7: A limited variety of foods and/or beverages from different cuisines and dietary requirements are widely available for purchase across all areas of the university<br>3: Foods and/or beverages from different cuisines and dietary requirements are available for purchase, but are not easily accessible across all areas of the university<br>0: No diversity in the types of cuisines available for purchase
**AVAIL4**	Do vending machines on campus contain predominantly healthier food and beverage options?	10: Generally vending machines across all areas of the university contain predominantly healthy foods and/or beverages<br>5: Some vending machines across the university contain predominantly healthy foods and/or beverages<br>2.5: Vending machines contain some healthy food and/or beverages options, but unhealthy options still predominate<br>0: Vending machines across the university contain little or no healthy foods and/or beverages
**AVAIL5**	Are healthier foods and beverages placed in the most prominent location within vending machines on campus?	10: Unhealthy foods and/or beverage are generally only displayed on the bottom shelves of vending machines<br>5: Unhealthy foods and/or beverage are mostly displayed on the bottom shelves of some or all vending machines<br>0: Unhealthy foods and/or beverages are displayed at main eye level within most vending machines
**AVAIL6**	Is there signage to help consumers understand the nutrient quality of foods and beverages within vending machines on campus?	5: Vending machines generally display nutrition signage that identifies healthy AND unhealthy foods and/or beverages<br>4: Vending machines generally display nutrition signage that identifies healthy foods and/or beverages only<br>2: Some vending machines display nutrition signage that identifies healthy and/or unhealthy foods and/or beverages<br>0: Vending machines do not display any nutrition signage

**AVAIL7**	Is a diverse range of culturally appropriate foods available within vending machines across campus?	5: A variety of foods and/or beverages from different cuisines are widely available for purchase in vending machines<br>2.5: Some cultural diversity in the foods and/or beverages available for purchase in vending machines<br>0: No cultural diversity in the foods and/or beverages available for purchase in vending machines
**AVAIL8**	Are self-catering facilities available for staff and student use across campus?	10: Adequately equipped self-catering facilities are readily accessible across all areas of the university<br>5: Self-catering facilities are available but have limited equipment OR are not readily accessible across all areas of the university<br>2.5: Self-catering facilities are available on some campuses but have limited equipment AND are not readily accessible within and/or across campuses<br>0: No evidence/No self-catering facilities are available
**AVAIL9**	Are healthier food options available for purchase at all times that campus facilities are open?	5: A variety of healthy snacks, meals and drinks are available for purchase across all areas of the university and at all times that campus facilities are open<br>3: A limited selection of healthy foods and drinks are available for purchase across all campuses at all times that campus facilities are open<br>1: Healthy foods and drinks are available for purchase at all times that campus facilities are open but selection and availability across the university is limited<br>0: No or very few healthy foods and drinks are available for purchase at all times that campus facilities are open
	**EQUITY (EQUI)**	
**EQUI1**	Does the university support food retailers to ensure the sale of healthy and sustainable food* is commercially viable?	10: Considerable support is given to food retailers to promote the sale of healthy and environmentally sustainable food that is affordable and commercially viable.<br>5: Some guidance is provided to food retailers to promote the sale of healthy and environmentally sustainable food that is affordable and commercially viable.<br>0: No evidence/No support or guidance for food retailers to promote the sale of healthy and environmentally sustainable food that is affordable and commercially viable.

**EQUI2**	Are healthy, low cost foods available for purchase across the campus?	10: A variety of healthy, affordable meals are widely available for purchase across all areas of the university<br>7: Healthy, affordable meals are available but selection is limited/mostly unhealthy OR not readily accessible across all areas of the university<br>3: Healthy, affordable meals are available but selection is limited/mostly unhealthy AND not readily accessible across all areas of the university<br>0: Healthy, affordable meals are not available for purchase across campus<br>Tip: Affordable meals may include having venues known for affordable food or having a 'cheap' option (normally between $5-10) at several food outlets
**EQUI3**	Is there a food relief program that predominantly provides healthy foods?	10: There is a food relief program that is widely accessible and predominantly provides healthy foods.<br>7.5: There is a food relief program but it is not widely accessible OR provides few healthy foods.<br>5: There is a food relief program but it is not widely accessible AND provides few healthy foods.<br>2.5: No food relief program on campus but there are services or information to direct individuals to local food relief programs.<br>0: No evidence/No food relief program or information about local programs available.
**ADVERTISING AND SPONSORSHIP (ADVER)**		
**ADVER1**	Are campuses free from advertising and marketing materials and activities that promote unhealthy foods or beverages?	10: All campuses are free from advertising and marketing materials and activities that promote unhealthy foods and/or beverages<br>5: There are some advertising and marketing materials and/or activities that promote unhealthy foods and/or beverages on campuses<br>2.5: Unhealthy food advertising and marketing materials and/or activities are common on campus grounds, but opportunities to reduce unhealthy food advertising are currently being explored<br>0: Unhealthy food advertising and marketing materials and/or activities are common on campus grounds
**ADVER2**	Are university events and organisations free from unhealthy food or beverage sponsorship?	10: All events and organisations on campuses are free from unhealthy foods and/or beverage sponsorship (including stalls, give-aways, promotion materials)<br>5: There is some unhealthy food sponsorship of events and/or organisations on campuses

		0: Unhealthy food and/or beverage sponsorship of events and/or organisations is common<br>N/A
	**EVENTS AND CATERING (CATER)**	
**CATER1**	Do policies and/or processes around catering and catered events promote the provision of healthy and environmentally sustainable foods* and beverages at catered events and meetings?	10: There is a comprehensive policy and/or process that promotes the provision of healthy and environmentally sustainable food at catered events and meetings.<br>7.5: There is a policy and/or process that promotes the provision of healthy and environmentally sustainable food at catered events and meetings, but it is not comprehensive and/or specific.<br>5: No current policy or process but planning and/or development underway.<br>0: No evidence/No current policy or process that promotes the provision of healthy and environmentally sustainable food at catered events and meetings.
**CATER2**	Do fundraising activities include and promote healthier food and beverage options?	10: All university fundraising activities include and promote healthier and more environmentally sustainable food and beverage options<br>5: Some campus fundraising activities include and promote healthier and more environmentally sustainable food and beverage options<br>0: No evidence/All or almost all fundraising activities do not include healthier and more environmentally sustainable food and beverage options<br>N/A: No events took place
**CATER3**	Is food provided at university-based, owned and/or run accommodation predominantly healthy?	10: Predominantly healthy foods and beverages are provided at all university-based, owned and/or run accommodation<br>7.5: Some healthy foods and beverages are provided at all university-based, owned and/or run accommodation<br>5: Some healthy foods and beverages are provided at a limited number of university-based, owned and/or run accommodation<br>0: No evidence/Predominantly unhealthy foods and beverages are provided at university-based, owned and/or run accommodation<br>N/A: No student accommodation or no food provided at student accommodation

PERSONAL AND COMMUNITY DEVELOPMENT (DEV)		
**DEV1**	Is there a community garden on campus?	10: There is a campus community garden readily accessible to all staff and students.<br>7: There is a campus community garden readily accessible to some staff and students.<br>3: There is no campus community garden, but there is information to direct individuals to local community gardens.<br>0: No campus community garden or information about local community gardens.
**DEV2**	Are there community programs to build skills in healthy and environmentally sustainable eating?	10: There is a program(s) to build skills in healthy and environmentally sustainable eating accessible to all staff and students.<br>7.5: There is a program(s) to build skills in healthy and environmentally sustainable eating accessible to some staff and students.<br>2.5: There are no programs to build skills in healthy and sustainable eating, but there is information to direct individuals to local community programs.<br>0: No evidence/No campus program or information about local programs to build skills in healthy and sustainable eating.
**DEV3**	Does the university encourage student and staff projects and research opportunities that enhance skills and knowledge related to healthy and sustainable food?	10: There is comprehensive support to encourage student and staff projects and research that aim to increase skills and knowledge related to healthy, equitable and environmentally sustainable food.<br>5: There is some support to encourage student and staff projects and research that aim to increase skills and knowledge related to healthy, equitable and environmentally sustainable food.<br>2.5: No student projects currently available or underway, but planning is in progress to make projects available to all students.<br>0: No evidence/No student projects available or underway related to healthy, equitable and environmentally sustainable food.
**DEV4**	Is there a food outlet on campus that champions best practice and tests innovative retail solutions to promote healthy, equitable and environmentally sustainable food?	5: There is a food outlet on campus that champions and evaluates innovative retail solutions to promote healthy, equitable and environmentally sustainable food<br>3: There is a food outlet on campus that champions innovative retail solutions to promote healthy, equitable and environmentally sustainable food, but impact of the strategies are not evaluated

		0: No evidence/No food outlet on campus that champions and evaluates innovative retail solutions to promote healthy, equitable and environmentally sustainable food
**DEV5**	Is information and/or training is available to all staff and students about healthy and sustainable eating?	5: Comprehensive training and/or information about healthy AND environmentally sustainable eating is available to all staff and students.<br>3: Comprehensive training and/or information about healthy OR environmentally sustainable eating is available to all staff and students.<br>1: Some training and/or information about healthy and/or environmentally sustainable eating is available to all staff and students.<br>0: No evidence/No training or information about healthy or environmentally sustainable eating for staff and students
**DEV6**	Is affordable nutrition counselling available to staff and students?	5: Affordable nutrition counselling is widely available across the university<br>4: Affordable nutrition counselling is available on some areas of the university<br>3: Nutrition counselling is available on campus<br>2: Nutrition counselling is not available on campus but there are services or information to direct individuals to local nutrition counselling<br>0: No evidence/No nutrition counselling or support available
	**ENVIRONMENTAL IMPACT (ENVIRO)**	
**ENVIRO1**	Is there strategy to reduce the volume of waste to landfill that acknowledges food waste?	10: There is a detailed and comprehensive strategy to reduce the volume of food waste to landfill<br>7.5: There is a strategy to reduce the volume of food waste to landfill but it is not detailed and/or does not specifically reference food waste<br>5: No current strategy but planning and/or development underway<br>0: No evidence/No strategy to reduce the volume of food waste to landfill
**ENVIRO2**	Are separate waste management bins provided for a minimum of landfill, recycling, and green waste?	10: Separated waste bins are provided across all areas of the university for a minimum of landfill, recycling and green waste<br>7.5: Separated waste bins are provided across some areas of the university for a minimum of landfill, recycling and green waste<br>5: Separated waste bins are provided for landfill and recycling only<br>0: Separated waste bins not provided
**ENVIRO3**	Does the university support redistribution/donation of foods through policy and/or coordination?	5: Food redistribution is actively supported and/or coordinated across the university<br>3: Food redistribution is actively supported and/or coordinated to a limited

		extent<br>1: Food redistribution is not actively conducted but is also not obstructed by institutional policy<br>0: No evidence/No redistribution/donation of campus food due to barriers imposed by institutional policy
**ENVIRO4**	Is there a policy that strongly discourages single-use plastics packaging and serving ware?	10: There is a detailed and comprehensive policy that strongly discourages single-use plastic packaging and serving ware for foods prepared and sold on campus<br>7.5: There is a policy that strongly discourages single-use plastic packaging and serving ware for foods prepared and sold on campus, but it is not comprehensive and/or specific<br>5: No current policy or commitment but planning and/or development underway<br>0: No evidence/No policy that discourages single-use plastic packaging and serving ware
**ENVIRO5**	Does the university have a strategy to reduce the volume of water used?	5: There is a detailed and comprehensive strategy to reduce campus water use that recognises the contribution of food environments<br>3: There is a strategy to reduce campus water use but it is not detailed and/or does not specifically reference food<br>1: No current strategy but planning and/or development underway<br>0: No evidence/No strategy to reduce campus water use
**ENVIRO6**	Does the university have a strategy to reduce energy emissions?	5: There is a detailed and comprehensive strategy to reduce campus energy emissions that recognises the contribution of food environments<br>3: There is a strategy to reduce campus energy emissions, but it is not detailed and/or does not specifically reference food<br>1: No current strategy but planning and/or development underway<br>0: No evidence/No current strategy to reduce campus energy emissions

FOOD RETAIL ENVIRONMENT		
**AVAILABILITY AND ACCESSIBILITY - RETAIL-LEVEL (AVAILR)**		
**AVAILR1**	Does the outlet sell predominantly healthy foods and beverages?	10: Predominantly healthy foods and beverages are available for purchase<br>5: A combination of both healthy and unhealthy foods and beverages are available for purchase<br>0: Predominantly unhealthy foods and beverages are available for purchase
**AVAILR2**	Does the outlet sell predominantly vegetarian or vegan foods and beverages?	10: Mostly vegetarian or vegan foods are available for purchase<br>7: A moderate selection of vegetarian or vegan foods are available for purchase<br>3: A very limited selection of vegetarian or vegan foods are available for purchase<br>0: No vegetarian or vegan foods available for purchase
**AVAILR3**	Are reduced portion sizes available for most items sold in the outlet?	10: Reduced portion sizes are available for all/most food and beverage items sold in the outlet<br>5: Reduced portion sizes are available for some food and beverage items sold in the outlet<br>0: Reduced portion sizes are not available for all/most food and beverage items sold in the outlet
**AVAILR4**	Are unhealthy food positioned away from prominent locations within the outlet?	10: Unhealthy foods and/or beverages are not placed in prominent locations within the outlet<br>5: Both healthy and unhealthy foods and/or beverages placed in prominent locations within the outlet<br>0: Predominantly unhealthy foods and/or beverages are placed in multiple prominent locations within the outlet
**PROMOTION (PROMO)**		
**PROMO1**	Is the outlet free from advertising that promotes unhealthy foods and beverages?	10: No advertisements for unhealthy foods or beverages within the outlet<br>5: Some advertisements for unhealthy foods and/or beverages within the outlet (e.g. on drinks fridges)<br>0: Multiple advertisements for unhealthy foods and/or beverages within the outlet

	**PRICE (PRICE)**	
**PRICE1**	Are food prices supportive of healthier choices?	10: The price of most foods and/or beverages in the retail outlet incentivise the purchase of healthy options over unhealthy options<br>7: The price of most foods and/or beverages in the retail outlet equally incentivises the purchase of unhealthy and healthy options<br>3: The price of some foods and beverages in the retail outlet incentivise unhealthy options over healthy options<br>0: The price of most food and beverages in the retail outlet incentivise unhealthy options over healthy options
**PRICE2**	Are food prices supportive of vegan and vegetarian options?	10: The price of most foods in the retail outlet incentivise the purchase of vegan or vegetarian options over meat-containing options<br>7: The price of most foods in the retail outlet equally incentivise the purchase of vegan, vegetarian and meat-containing options<br>3: The price of some foods in the retail outlet incentivise meat-containing options over vegan or vegetarian options<br>0: The price of most food in the retail outlet incentivise meat-containing options over vegan or vegetarian options
**PRICE3**	Is the outlet free from price promotions that encourage larger portion sizes?	5: There are no price promotions that encourage larger portion sizes within the outlet<br>0: There are price promotions that encourage larger portion sizes within the outlet
**PRICE4**	Are unhealthy foods excluded from discounted 'meal deals'?	10: Unhealthy foods and/or beverages are not included in any discounted 'meal deals'<br>7: Discounted 'meal deals' offer a choice of healthy or unhealthy options but healthy options are provided as the default<br>5: Discounted 'meal deals' offer a choice of healthy or unhealthy options<br>0: All/most discounted 'meal deals' include predominantly unhealthy foods and/or beverages<br>N/A: No meal deals offered

	**INFORMATION (INFO)**	
**INFO1**	Is there clear signage that allows consumers to understand the nutrient quality of foods for sale?	10: Interpretive nutrition signage is displayed that identifies healthy AND unhealthy# foods and/or beverages<br>7: Interpretive nutrition signage is displayed that identifies healthy# foods and/or beverages only<br>3: Reductive nutrition signage is displayed (i.e. information only)<br>0: No nutritional signage displayed within the outlet
**INFO2**	Is there clear signage that allows consumers to understand the environmental sustainability of foods for sale?	This indicator is currently not being assessed
**INFO3**	Is there clear signage to identify foods that meet different dietary requirements?	10: Signage to identify foods and/or beverages that meet a wide variety of different dietary requirements is displayed<br>7: Signage to identify foods and/or beverages that meet several different dietary requirements is displayed<br>3: Signage to identify foods and/or beverages that meet a limited number of dietary requirements is displayed<br>0: No signage to identify foods and/or beverages that meet different dietary requirements
	**ENVIRONMENTAL IMPACT - RETAIL-LEVEL (ENVIROR)**	
**ENVIROR1**	Does the outlet only use reusable, recyclable or compostable packaging for foods prepared and sold?	10: Only reusable packaging is used for foods and/or beverages prepared within the outlet<br>7.5: Only reusable/recyclable/compostable packaging is used for foods and/or beverages prepared within the outlet<br>5: Mostly reusable/recyclable/compostable packaging is used for foods and beverages prepared within the outlet<br>2.5: Both reusable/recyclable/compostable and single-use packaging is used within the outlet<br>0: Predominantly single-use plastic packaging is used within the outlet
**ENVIROR2**	Does the outlet encourage the use of BYO or returnable packaging?	10: Financial incentives are offered to encourage BYO or returnable packaging<br>7: Signage is displayed to encourage BYO or returnable packaging<br>3: No encouragement, but BYO/returnable packaging is accepted<br>0: BYO packaging not accepted
**ENVIROR3**	Does the outlet have a waste monitoring and reduction program in place?	10: There is a comprehensive waste monitoring and reduction program in place

	5: No formal program in place, but some waste monitoring and reduction activities are conducted within the outlet<br>0: Waste monitoring or reduction activities are not conducted within the outlet

* Healthy and sustainable foods are foods that are classified as part of the 'five food groups' (products recommended for regular consumption) as part of the Australian Dietary Guidelines (12). These foods are generally lower in added sugar, sodium and harmful fats. They are mostly plant-based and contain less animal-based products. In addition, seasonal and local produce that is minimally processed, products with minimal packaging and that have not been imported by air, are typically better for the environment. Healthy diets include limited consumption of unhealthy ('discretionary') foods that are often 'ultra-processed' and high in energy and added sugar, sodium and/or harmful fats (12-14).

File S1. Questionnaire assessor evaluation

1. **Assessor and university details**

1a) Please state your university name.

1b) When did you conduct the audit?

Start date (approx.) ___________________________

End date (approx.) _____________________

1c) What degree are you currently studying (if relevant)?

1d) Have you undertaken any nutrition units as part of this or a previous degree?

2. Uni-Food tool online training

2a) Did you find the Uni-Food tool training session useful? Five-point Likert scale (1=very useful, 5=not useful at all)

2b) Do you believe the Uni-Food tool training session provided sufficient information/detail of all the aspects of the Uni-Food tool and how to complete your university audit?

☐ Yes

☐ No *If no, please describe what further information/detail you would have liked to receive in the training session*

2c) Do you have any suggestions to improve the Uni-Food tool training session?

3. Desk audit component of Uni-Food

3a) Approximately how many hours did it take you to complete the **desk audit** of your university?

3b) Were there any indicators or indicator descriptions in the **desk audit** that you found difficult to understand?

☐ Yes *If yes, please describe which indicators were difficult to understand.* _________________

☐ No

3c) How easy/difficult did you find it to complete the **desk audit** of your university? Five-point Likert scale scale (1=very easy, 5=very difficult)

3d) Did you face any difficulties with the **desk audit** of your university?

☐ Yes *If yes, please describe what difficulties you faced.* _________________

☐ No

4. Campus food environment audit component of Uni-Food

4a) Approximately how many hours did it take you to complete the **campus food environment audit** of your university?

4b) Were there any indicators or indicator descriptions in the **campus food environment audit** that you found difficult to understand?

☐ Yes *If yes, please describe which indicators were difficult to understand.* ________________

☐ No

4c) How easy/difficult did you find it to complete the **campus food environment audit** of your university? Five-point Likert scale (1=very easy, 5=very difficult)

4d) Did you face any difficulties when completing the **campus food environment audit** of your university?

☐ Yes *If yes, please describe what difficulties you faced.* ________________

☐ No

5. Campus retail audit component of Uni-Food

5a) Approximately how many hours on average did it take you to complete the **campus retail audit** for **each** retailer at your university?

5b) Across all the campuses that you audited **how many retailers** did you assess at your university?

5c) Approximately how many hours did it take **in total** for you to complete the **campus retail audit** for all the retailers at your university (please include all campuses)?

5d) Were there any indicators in the **campus retail audit** that you found difficult to understand?

☐ Yes *If yes, please describe which indicators were difficult to understand.* ________________

☐ No

5e) How easy/difficult did you find it to complete the **campus retail audit** of your university? Five-point Likert scale (1=very easy, 5=very difficult)

5f) Did you face any difficulties when conducting the **campus retail audit** of your university?

☐ Yes *If yes, please describe what difficulties you faced.* ________________

☐ No

6. Overall experience with the Uni-Food tool

6a) In total, how many hours did it take you to complete the audit of your university? Please take into consideration all contact you had with the Deakin University research team, the appropriate university contacts and meetings with your supervisors.

6b) Do you believe the indicators in the Uni-Food tool covered all relevant areas of the university food environment related to healthy and sustainable food environments?

☐ Yes

☐ No *If no, please describe what areas could be added to the Uni-Food tool:* ________________

6c) Do you believe the Uni-food tool is an effective tool to help assess university food environments? Five-point Likert scale (1=very ineffective, 5=very effective)

6d) Do you have any suggestions to improve the Uni-Food tool?

File S2. Results assessor evaluation

Evaluation feedback

All six assessors completed the evaluation survey and rated the Uni-Food training received as extremely useful (Table S6) especially the demonstrated examples of how to use the Uni-Food tool. No assessors had any suggestions of further indicators that should be added to the tool. Most students reported the Uni-Food tool to be an effective tool to assess the university food environment.

The median amount of time reported by assessors required to complete the entire audit was 45 hours. This range was large as evidenced by an inter quartile range (IQR) between 37.5-80.0 hours. This is most likely due to the different number of retailers (10-21) across the three universities and the variability in size of the campuses. Of the three components of the audit, the desk audit required the least amount of time to complete (median=7.5 hours, IQR: 5.6-7.5 hours), followed by the campus audit (median=13.5 hours, IQR: 5.1-22.0 hours). The time taken to complete the retailer audit was slightly longer with a median of 14.0 hours (IQR: 11.5-14.0).

Assessors rated completing the desk audit as the most difficult of the three components of the Uni-Food audit, due to the appropriate information being challenging to find online, resulting in assessors having to liaise with members of the campus facilities staff. Most assessors believed the campus audit was easy to complete, while half believed the retail audit was easy to complete.

Suggestions provided to improve the Uni-Food tool included: changing the wording of some indicator descriptions and scoring criteria; and enhancing the usability of the tool itself, through greater use of online technologies to assist with data collection. The feedback received was used to the amend the tool to resolve ambiguities.

Table S4. Assessors' (n = 6) evaluation of the Uni-Food tool and training session.

Item	Response
Did you find the Uni-Food training session useful?	Extremely useful=6 (100%)<br>Very useful=0<br>Moderately useful=0<br>Slight useful=0<br>Not useful at all=0
Do you believe the indicators covered all relevant areas of the university food environment related to healthy and sustainable food environments?	Yes=6 (100%)<br>No=0
Do you believe the Uni-Food tool is an effective tool to help assess university food environments?	Very effective=5 (67%)<br>Effective=0<br>Neither effective nor ineffective=0<br>Ineffective=0<br>Very ineffective=1 (17%)
Total amount of time (hours): Median (IQR)	45 hours (37.5-80.0)
Desk audit time taken (hours): Median (IQR)	7.50 (5.63-7.50)
Campus audit time taken (hours): Median (IQR)	13.5 (5.13-22.00)
Retailer audit time taken (hours): Median (IQR)	Per retailer: 1.00 (0.81-1.00)<br>Total: 14.00 (11.5.-14.00)
How easy/difficult did you find it to complete the **desk audit** of your university?	Extremely easy=0<br>Somewhat easy=0<br>Neither easy nor difficult=3 (50%)<br>Somewhat difficult=3 (50%)<br>Extremely difficult=0
How easy/difficult did you find it to complete the campus **audit** of your university?	Extremely easy=0<br>Somewhat easy=5 (83%)<br>Neither easy nor difficult=1 (17%)<br>Somewhat difficult=0<br>Extremely difficult=0
How easy/difficult did you find it to complete the **retailer audit** of your university?	Extremely easy=0<br>Somewhat easy=3 (50%)<br>Neither easy nor difficult=1 (17%)<br>Somewhat difficult=1 (17%)<br>Extremely difficult=1 (17%)

References

1. Fair Food Challenge. Fair food challenge: what could a fair food university look like?2016. Available from: https://fairfoodchallenge.com/.
2. Roy R, Hebden L, Kelly B, De Gois T, Ferrone EM, Samrout M, et al. Description, measurement and evaluation of tertiary-education food environments. British Journal of Nutrition. 2016;115(9):1598-606.
3. Swinburn B, Vandevijvere S, Kraak V, Sacks G, Snowdon W, Hawkes C, et al. Monitoring and benchmarking government policies and actions to improve the healthiness of food environments: a proposed Government Healthy Food Environment Policy Index. Obesity Reviews. 2013;14(S1):24-37.
4. Dooris M, Cawod J, Doherty S, Powell S. Healthy universities: concept, model and framework for applying the healthy settins approach within higher education in England. Final project report2010. Available from: https://healthyuniversities.ac.uk/.
5. The Culinary Institute of America. Menus of Change. Principles of healthy, sustainable menus 2021. Available from: https://www.menusofchange.org/principles-resources/moc-principles/.
6. Saelens BE, Glanz K, Sallis JF, Frank LD. Nutrition Environment Measures Study in Restaurants (NEMS-R): Development and Evaluation. American Journal of Preventive Medicine. 2007;32(4):273-81.
7. Lo BKC, Minaker L, Chan ANT, Hrgetic J, Mah CL. Adaptation and Validation of a Nutrition Environment Measures Survey for University Grab-and-Go Establishments. Canadian Journal of Dietetic Practice and Research. 2015;77(1):17-24.
8. Lee KM, Marcinow ML, Minaker LM, Kirkpatrick SI. The Healthfulness of Eateries at the University of Waterloo: A Comparison across 2 Time Points. Canadian Journal of Dietetic Practice and Research. 2019;81(2):72-9.
9. Horacek TM, Simon M, Dede Yildirim E, White AA, Shelnutt KP, Riggsbee K, et al. Development and Validation of the Policies, Opportunities, Initiatives and Notable Topics (POINTS) Audit for Campuses and Worksites. International Journal of Environmental Research and Public Health. 2019;16(5).
10. The Association for the Advancement of Sustainability in Higher Education. STARS - technical manual2019. Available from: https://stars.aashe.org/resources-support/technical-manual/.
11. Freedman MR. Development, Evaluation, and Validation of Environmental Assessment Tools to Evaluate the College Nutrition Environment. Journal of American College Health. 2010;58(6):565-8.
12. National Health and Medical Research Council. Australian Dietary Guidelines. Canberra: Australian Government, 2013.
13. Macdiarmid JI, Kyle J, Horgan GW, Loe J, Fyfe C, Johnstone A, et al. Sustainable diets for the future: can we contribute to reducing greenhouse gas emissions by eating a healthy diet? The American journal of clinical nutrition. 2012;96(3):632-9.
14. Willett W, Rockström J, Loken B, Springmann M, Lang T, Vermeulen S, et al. Food in the Anthropocene: the EAT–Lancet Commission on healthy diets from sustainable food systems. The Lancet. 2019;393(10170):447-92.
